# Skin Carotenoid Scores Reflect Immune Status among Children Living with HIV in Tanzania

**DOI:** 10.4269/ajtmh.26-0169

**Published:** 2026-09-03

**Authors:** Wilbert Mbuya, Thobias Boniphace, Michael Mwagala, Peter Agrea, Nazareth M. Mbilinyi, Nancy E. Moran, Andrew R. DiNardo, Mkunde Chachage

**Affiliations:** ^1^ National Institute for Medical Research–Mbeya Medical Research Center (NIMR-MMRC), Mbeya, Tanzania;; ^2^ Global Tuberculosis Program, Texas Children’s Hospital, Immigrant and Global Health, Department of Pediatrics, Baylor College of Medicine, Houston, Texas, USA;; ^3^ Department of Microbiology and Immunology, University of Dar es Salaam Mbeya College of Health and Allied Sciences (UDSM-MCHAS), Mbeya, Tanzania;; ^4^ Mbeya Zonal Referral Hospital, Mbeya, Tanzania;; ^5^ Children’s Nutrition Research Center, Department of Pediatrics, Baylor College of Medicine, Houston, Texas, USA

## Abstract

Many individuals living with HIV experience persistent inflammation despite antiretroviral treatment, increasing their risk for cardiovascular diseases, cognitive decline, and other noncommunicable diseases. Carotenoids can inhibit this inflammation by reducing lipid peroxidation. Carotenoid intake and plasma concentrations can be approximated using a noninvasive cutaneous spectrophotometer. This study quantified skin carotenoid levels in Tanzanian children ages 5 to 15 years old (*N* = 185) both with and without HIV. Over 95% had low to moderate skin carotenoid scores (<400 units). In children living with HIV, higher viral loads were linked to lower skin carotenoid scores (rho = −0.57, *P* <0.001), whereas higher CD4 counts correlated with increased skin carotenoid scores (rho = 0.52, *P* <0.001). Overall, carotenoid levels were low and associated with immune status, suggesting that elevated carotenoid scores correlate with lower inflammation in children living with HIV. Future studies investigating dietary carotenoid interventions to support immune function are warranted.

## INTRODUCTION

Chronic infectious diseases, such as HIV and tuberculosis, are associated with unresolved long-term residual inflammation, despite successful antimicrobial treatment. This inflammation has been linked to elevated risk for cardiovascular diseases, cancers, and accelerated cognitive decline.^1^^,^^2^ Inflammation is propagated by the combination of continued metabolic perturbations and a failure to reduce an oxidized intracellular environment in immune cells.^3^ A potential solution to lower inflammation and thereby, avert long-term consequences of inflammation is to consume foods that enhance plasma-reducing capacity.^4^^–^^6^

Carotenoids are compounds with strong singlet oxygen-reducing capacity; they are usually found in red, yellow, orange, and dark green fruits and vegetables, such as carrots, sweet potatoes, pumpkins, kale, papaya, and yams.^7^ Plausibly, the unreduced pro-oxidant intracellular environment leads to lipid peroxidation of intracellular membranes. Products of lipid peroxidation, such as malondialdehyde and 4-hydroxynonenal, fuel cellular inflammatory pathways, thereby leading to chronic inflammation.^8^^,^^9^ Approximately 1 month of carotenoid supplementation through either diet or pill supplements has been demonstrated to lower inflammation.^4^^–^^6^ However, the cited studies did not explore further either the duration of elevated carotenoid levels or the duration of sustained lowered inflammation. It is, therefore, important to monitor nutrition biomarkers, such as carotenoids, through noninvasive, field-tested, and affordable methods. The Veggie Meter^®^ (Longevity Link Corporation, Salt Lake City, UT, USA), which is characterized by easy rollout, cost-effectiveness, and noninvasiveness, measures combined skin carotenoid (SC) concentrations in real time using reflection spectroscopy. It has shown strong correlations with plasma carotenoid levels among people from diverse races but has not yet been deployed in Africa,^10^ where logistics, culture, and nutrition differ from Europe and America, the regions where the device was piloted. Previous studies have shown that Veggie Meter SC scores strongly correlate with total plasma carotenoids (*R* = 0.81; *P* <0.001).^11^

The objective of this pilot study was to evaluate cutaneous carotenoid scores in children living with HIV and children without HIV and to further examine the relationship between HIV-associated immune parameters (CD4 T-cell counts and HIV viral load) and SC score in Tanzanian children living in the southern highlands.

## MATERIALS AND METHODS

### Study population.

Convenience sampling was used to recruit 185 male and female volunteers ages 5 to 15 years old at the pediatric wing of the outpatient department of the Mbeya Zonal Referral Hospital, a tertiary facility serving the Southern Highland Zone of Tanzania. Parents/guardians and children were approached in hospital waiting bays and invited to participate after they had completed their primary hospital visit. Eligibility was assessed for willing participants. Only participants ages 5 to 15 years of age without mental disability were enrolled into the study. All participants were enrolled into the cross-sectional study between August and October 2025.

### Skin carotenoid score.

**Figure 1.**
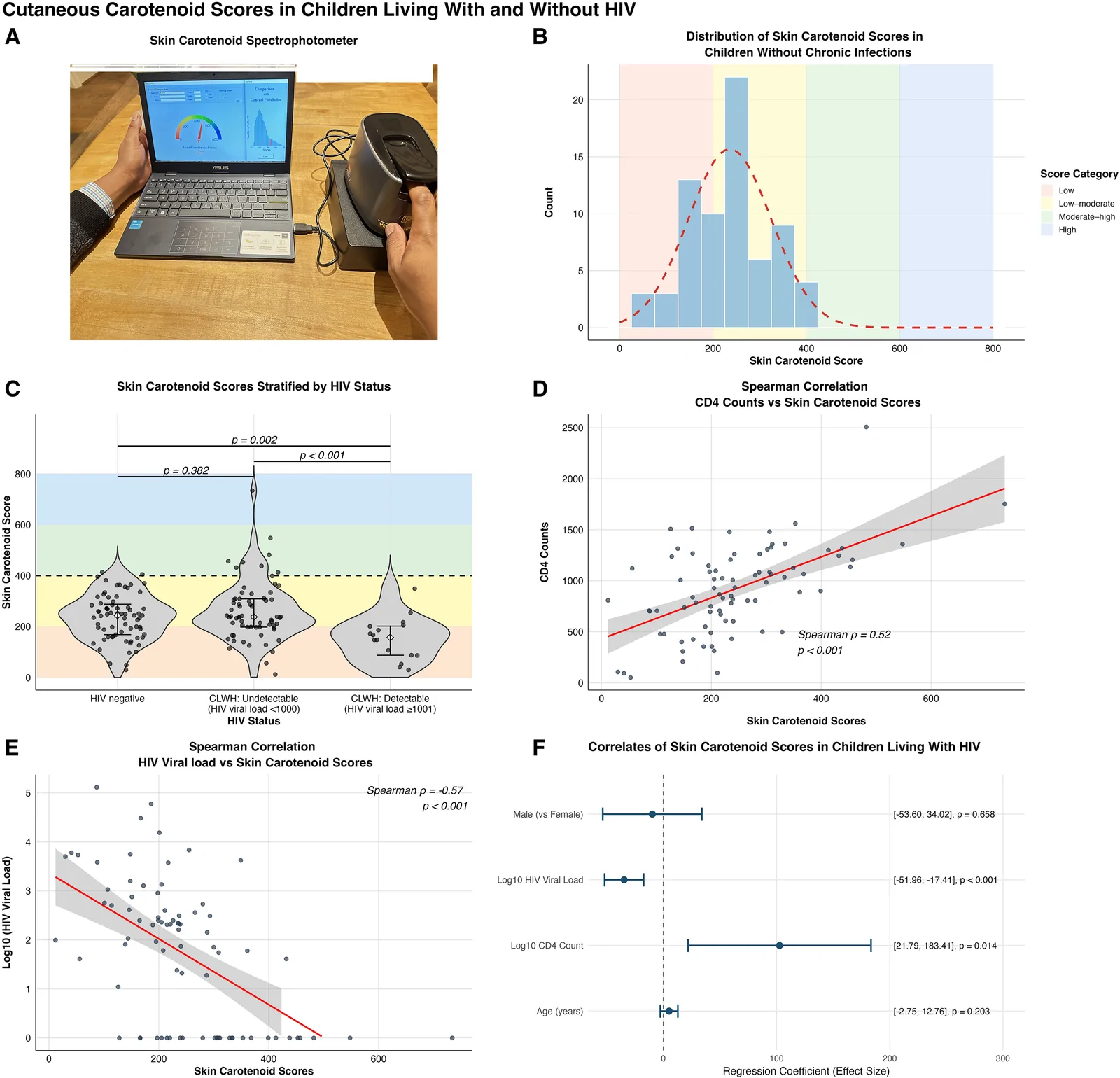
Skin carotenoid scores among Tanzanian children with and without HIV. (**A**) Representative image of the cutaneous carotenoid spectrophotometer (Veggie Meter). (**B**) Gaussian distribution of skin carotenoid scores in children without chronic infections. (**C**) Skin carotenoid scores categorized by HIV status and plasma HIV viral load. (**D**) Relationship between skin carotenoid scores and CD4 T-cell count. (**E**) Association between skin carotenoid scores and HIV viral load. (**F**) Linear regression analysis evaluating the influence of age, sex, CD4 T-cell count, and plasma HIV viral load among children living with HIV (CLWH).

Skin carotenoid quantification was implemented using a real-time noninvasive reflection spectrophotometer^11^ (Veggie Meter) (Figure 1A). Consented participants placed their ring finger of their nondominant hand onto the lens inside the machine. Measurements were taken regardless of mealtimes because the test measures the cumulative amount of total carotenoid present in the skin at the time of quantification. All scores were measured in triplicate. Carotenoid scores were displayed and recorded in real time on a computer connected with the spectrophotometer (Figure 1A). According to the manufacturer’s guidelines, SC scores are interpreted as follows. Below 200 units are classified as low, 200 to 399 units are classified as low to moderate, 400 to 599 units are classified as moderate to high, and 600 units or above are classified as high level.

### Questionnaire.

A questionnaire to collect information on age, sex, and chronic disease status, including HIV, diabetes, tuberculosis, sickle cell, and asthma, was administered to all participants. CD4 counts and HIV viral load data were collected from clinic cards of participants living with HIV.

## STATISTICAL ANALYSES

The Mann–Whitney *U* test was used to determine if SC levels differed by HIV status. The Spearman correlation test was used to determine the relationship between HIV plasma viral load and CD4 counts with SC levels. A linear regression model was applied to determine the degree to which age, sex, viral load, and CD4 counts were associated with SC scores in children living with HIV. The regression model was adjusted for all covariates included in the model. Missing data were omitted from the relevant statistical analyses, and only complete case observations were included. No imputation procedures were applied. For all statistical tests, a *P*-value of less than 0.05 was regarded as significant. Statistical analyses were conducted using R v. 4.4.3 (R Foundation, Vienna, Austria).

## RESULTS

A total of 185 participants ages 5 to 15 years old were recruited from the outpatient department of the pediatric department of the Mbeya Zonal Referral Hospital. Eighty-two participants were living with HIV, whereas 103 were not living with HIV. Plasma HIV viral load, CD4 counts, and chronic infection status are displayed in Table 1.

**Table 1:** Baseline characteristics for children enrolled in the study (*N* = 185)

Characteristic	CLWoH (*n* = 103)	CLWH (*n* = 82)	*P*-Value
Age (years)	8.0 (6.0, 11.0)*	13.0 (10.0, 14.0)*	<0.001^†^
Sex			0.2^‡^
Female	40 (39%)	39 (48%)	
Male	63 (61%)	43 (52%)	
CD4 counts (cells/*µ*L)		932 (661, 1,238)*	
Plasma HIV viral load (copies/mL)		95 (0, 504)*	
Chronic diseases other than HIV			<0.001^§^
Asthma	2 (2%)	0 (0%)	
Diabetes	1 (1.0%)	0 (0%)	
Sickle cell	21 (20%)	0 (0%)	
Tuberculosis	1 (1.0%)	0 (0%)	
Other^‖^	8 (8%)	0 (0%)	
None	70 (68%)	83 (100%)	

CLWH = children living with HIV; CLWoH = children living without HIV. Numbers are *n* (%) unless otherwise noted.

* Median (quarter 1, quarter 3).

^†^ Mann–Whitney *U* test.

^‡^ Pearson χ^2^ test

^§^ Fisher exact test.

^‖^ This includes long-term medical conditions lasting at least 3 months, excluding asthma, diabetes, sickle cell disease, and tuberculosis. This category includes chronic neurological disorders (e.g., epilepsy and cerebral palsy), congenital or genetic conditions (e.g., congenital heart disease), chronic kidney or liver disease, and long-term developmental or physical disabilities requiring ongoing medical care.

We established baseline SC scores for children enrolled in the study by evaluating data from children without any chronic diseases (*n* = 70). Analyses indicated that more than 95% (*n* = 68/70) of the children had low to moderate SC scores (less than 400 units) (Figure 1B). When the studied population was stratified by HIV status and plasma HIV viral load, participants with viral loads higher than 1,000 had the lowest SC scores compared with those with lower viral loads or without HIV (Figure 1C). Notably, there was a positive correlation between SC scores and CD4 counts (Figure 1D) (Spearman rho = 0.52, *P* <0.001) and an inverse correlation between SC scores and plasma HIV viral load (Figure 1E) (Spearman rho = −0.57, *P* <0.001). Linear regression adjusted for age and sex on SC scores among children living with HIV confirmed that SC scores correlated with CD4 counts and plasma HIV viral load; an increase of one log_10_ for HIV viral load corresponded to a decrease in 35 units of SC scores, whereas an increase in one log of CD4 counts corresponded to an increase in 103 units of SC scores (Figure 1F) (*P* <0.05 for both).

## DISCUSSION

Carotenoids are a family of lipid soluble dietary compounds with strong capacity to reduce cellular oxidative stress, thereby halting the lipid peroxidation that drives persistent inflammation and the subsequent increased risk for noncommunicable diseases, such as cardiovascular diseases and accelerated cognitive decline.^4^^,^^5^^,^^12^

This report demonstrated that >95% of children enrolled in this study had low to moderate SC levels, and it demonstrated that the level of immune suppression (increased viral load and reduced CD4 counts) was significantly correlated to SC levels. Previous studies have demonstrated that HIV viral load levels are significantly associated with systemic immune inflammation.^13^ One can, therefore, postulate that in levels of severe immune suppression (a higher inflammatory state), carotenoid are consumed by the body’s cellular machinery at a rate faster than they can be replaced. Replacing depleted carotenoids along with disease-specific treatment has been demonstrated to be an effective treatment in cardiovascular diseases,^7^ rheumatologic diseases,^14^ and obesity,^4^ and therefore, it could potentially avert future risk associated with unresolved long-term inflammation among people living with chronic infectious diseases, such as HIV and tuberculosis.

Monitoring children’s nutrition accurately has many challenges, including recall bias. Furthermore, testing for specific nutrients often involves invasive and expensive procedures. In contrast, using a portable cutaneous spectrophotometer to quantify carotenoid levels offers a real-time, point-of-care, noninvasive, and reliable means to track carotenoid-based interventions. Study limitations included hospital-based recruitment because of funding constraints, thereby limiting our participants to those attending the hospital for either an illness or a routine clinical visit. However, we thoroughly interviewed parents and documented chronic diseases. Additionally, a cross-sectional study limited analysis of SC scores over time. Nonetheless, this study establishes the feasibility of implementing portable cutaneous carotenoid spectrophotometers in an African setting and in African children. Future studies should expanded to the general population, and they should evaluate if this point-of-care device can be used to guide increased carotenoid consumption, decrease inflammation, and mitigate infection-induced noncommunicable diseases. Additionally, future studies should explore in a longitudinal analysis carotenoids level after intervention and if the intervention has long-lasting effects on inflammation.

## CONCLUSION

This brief report demonstrated that carotenoid deficiencies are associated with immune deficiency in the children studied, which may predispose them to a higher risk of developing noncommunicable or inflammatory diseases conditions.
